# Jasmonates: Emerging Players in Controlling Temperature Stress Tolerance

**DOI:** 10.3389/fpls.2015.01129

**Published:** 2016-01-06

**Authors:** Manvi Sharma, Ashverya Laxmi

**Affiliations:** National Institute of Plant Genome ResearchNew Delhi, India

**Keywords:** jasmonates, abiotic stresses, heat, cold, signaling

## Abstract

The sedentary life of plants has forced them to live in an environment that is characterized by the presence of numerous challenges in terms of biotic and abiotic stresses. Phytohormones play essential roles in mediating plant physiology and alleviating various environmental perturbations. Jasmonates are a group of oxylipin compounds occurring ubiquitously in the plant kingdom that play pivotal roles in response to developmental and environmental cues. Jasmonates (JAs) have been shown to participate in unison with key factors of other signal transduction pathway, including those involved in response to abiotic stress. Recent findings have furnished large body of information suggesting the role of jasmonates in cold and heat stress. JAs have been shown to regulate C-repeat binding factor (CBF) pathway during cold stress. The interaction between the integrants of JA signaling and components of CBF pathway demonstrates a complex relationship between the two. JAs have also been shown to counteract chilling stress by inducing ROS avoidance enzymes. In addition, several lines of evidence suggest the positive regulation of thermotolerance by JA. The present review provides insights into biosynthesis, signal transduction pathway of jasmonic acid and their role in response to temperature stress.

## Introduction

Adequate perception, amalgamation and transduction of signals are obligatory for the growth and development of an organism. Plant hormones are a group of structurally diverse signal molecules that organize all cellular processes, consequently ensuring an effectual developmental plan and rationalized use of resources. Plant hormones therefore act as middlemen in the transmittance of information from the environment to the organism. The study of plant hormones is centuries old when the 5 classical hormones auxin, cytokinin, ABA, GA, and ethylene were described. However, over the last decade many “non-traditional” plant growth regulators have been described. These include highly diverse group of oxidized compounds, collectively known as oxylipins. Oxylipins execute diverse functions ranging from developmental processes to stress responses in plants (Andersson et al., 2006). Plant oxylipins can be produced either enzymatically by LIPOXYGENASES (LOXs) or α-DIOXYGENASES (α-DOXsas) or non-enzymatically by autoxidation of polyunsaturated fatty acids (Göbel and Feussner, 2009). One of the well characterized examples of oxylipins is Jasmonates (JAs).

The history of jasmonates is very old and dates back to 1960 when Demole successfully characterized methyl jasmonate (MeJA) from jasmine flower *Jasminum grandiflorum*. (Demole et al., 1962). Like many small esters, MeJA is volatile and has a sweet fragrance. Jasmonic acid (JA), on the other hand was isolated from fungal culture filtrate of *Lasiodiplodia theobromae* (Aldridge et al., 1971). JAs modulate many essential roles in plant development ranging from germination to vegetative growth to senescence. The role of JAs in dicotyledons such as tomato and *Arabidopsis* is well known, they are directly entailed in a number of physiological processes like stamen and trichome development, vegetative growth, cell cycle regulation, senescence, anthocyanin biosynthesis regulation, fruit ripening, cell cycle regulation (Parthier, 1991; Koda et al., 1992; Sembdner and Parthier, 1993; Creelman and Mullet, 1995, 1997; Koda, 1997; Wasternack and Hause, 2002; Browse, 2005; Wasternack, 2007; Balbi and Devoto, 2008; Pauwels et al., 2008; Zhang and Turner, 2008; Reinbothe et al., 2009; Yoshida et al., 2009). In addition, JAs activate plant defense mechanisms in response to insect-driven wounding, pathogen attack, and environmental stress, such as low temperature, salinity, heavy metal toxicity (Creelman and Mullet, 1997; Wasternack, 2007; Howe and Jander, 2008; Browse, 2009; Pauwels and Goossens, 2011). Studies in monocots have also confirmed the indispensible role of JAs in reproductive bud initiation and elongation, sex determination, leaf senescence and responses to the attack by pathogens and insects (Engelberth et al., 2004; Tani et al., 2008; Acosta et al., 2009; Yan et al., 2012).

## Biosynthesis of jasmonates

JAs are biosynthesized by the sequential action of enzymes present in plastid, peroxisome and cytoplasm (Feussner and Wasternack, 2002). JA biosynthesis is initiated by the release of α-LINOLENIC ACID (α-LeA) (18:3) from chloroplast membranes by PHOSPHOLIPASE1 (PLA1) to generate JA substrate (Vick and Zimmerman, 1983). α-LeA liberation is followed by the incorporation of molecular oxygen by the lipoxygenase family enzyme, LINOLEATE OXYGEN OXIDOREDUCTASE (13-LOX) at carbon atom 13 of the substrate forming 13*S*-HYDROPEROXY-(*9Z*,11*E*,15)-OCTADECATRIENOIC ACID (13-HPOT). 13-HPOT undergoes dehydration by the ALLENE OXIDE SYNTHASE (AOS) to form cis (+)12-OXO-PHYTODIENOIC ACID (OPDA) (Turner et al., 2002; Devoto and Turner, 2003; Wasternack, 2007). Similarly, LOX, AOS and AOC together catalyze hexadecatrienonic acid (C16:3) to form dinor-OPDA (dnOPDA). OPDA and dnOPDA are further transported to peroxisome via transporter COMATOSE (CTS1) (Theodoulou et al., 2005), wherein, they are reduced to OXOPHYTOENIC ACID (OPC-8) and 12-OXOPHYTOENIC ACID (OPC-6), respectively by OPDA reductase 3 (OPR3). OPDA, dnOPDA, OPC8 and OPC6 are activated by the ACYL-COENZYME A SYNTHETASES to form CoA esters, so that the carboxylic acid side chains can be shorted by two or three rounds of β-oxidation by ACYL-COA OXIDASE (ACX), a MULTIFUNCTIONAL PROTEIN (MFP), and L-3-KETOACYL COA THIOLASE (KAT) (Schneider et al., 2005). Jasmonoyl-CoA, the final product of the β-oxidation reactions, is cleaved by THIOESTERASE (TE) to form cis-7-iso-jasmonic acid [(+)-7-iso-JA]. It is then catabolized further by JA CARBOXYL METHYLTRANSFERASE (JMT) to form volatile counterpart MeJA. MeJA ESTERASE (MJE) in turn converts MeJA back to JA. The reversible conversion between JA and Jasmonoyl-isoleucine (JA-Ile) is catalyzed by a JASMONATE AMINO ACID SYNTHETASE (JAR1).

## Metabolic fate of JA

There are plethora of jasmonate compounds. JA goes through several biochemical modifications (Sembdner and Parthier, 1993; Koch et al., 1997; Seo et al., 2001; Staswick and Tiryaki, 2004; Swiatek et al., 2004). JA, Cis Jasmone (CJ), MeJA and JA-Ile have some biological activity in plants (Wasternack, 2007; Fonseca et al., 2009a). CJ, a volatile counterpart of JA is biologically active and is released in response to herbivory and insect driven attack. Transcriptome analysis data of CJ treated *Arabidopsis* plants provided insights into a *COI1*-independent CJ signaling (Matthes et al., 2010).

Miersch and co-workers in 2008 reported the presence of high levels of 12-OH-JA, 12-HSO4-JA, and 12-O-Glc-JA in immature seeds and leaves of *Glycine max* and *Zea mays* (Miersch et al., 2008). The role of jasmonates (JAs) 12-OH-JA, 12-HSO4-JA, and 12-O-Glc-JA in sex determination has been studied in *Zea mays* (Acosta et al., 2009; Browse, 2009). JA, MeJA, and CJ are considered useful tools in anti-cancer therapy as they are known to induce cell death by mitochondria perturbation and subsequent release of cytochrome oxidase (Kim et al., 2004; Rotem et al., 2005). Michelet et al. (2012) reported the anti-aging potential of tetra-hydro-jasmonic acid in humans. Tetra-hydro-jasmonic acid is known to increase the synthesis of hyaluronic acid by increasing the expression of hyaluronase synthase 2 and hyaluronase synthase 3.

## Jasmonic acid perception and signaling

**Figure 1:**
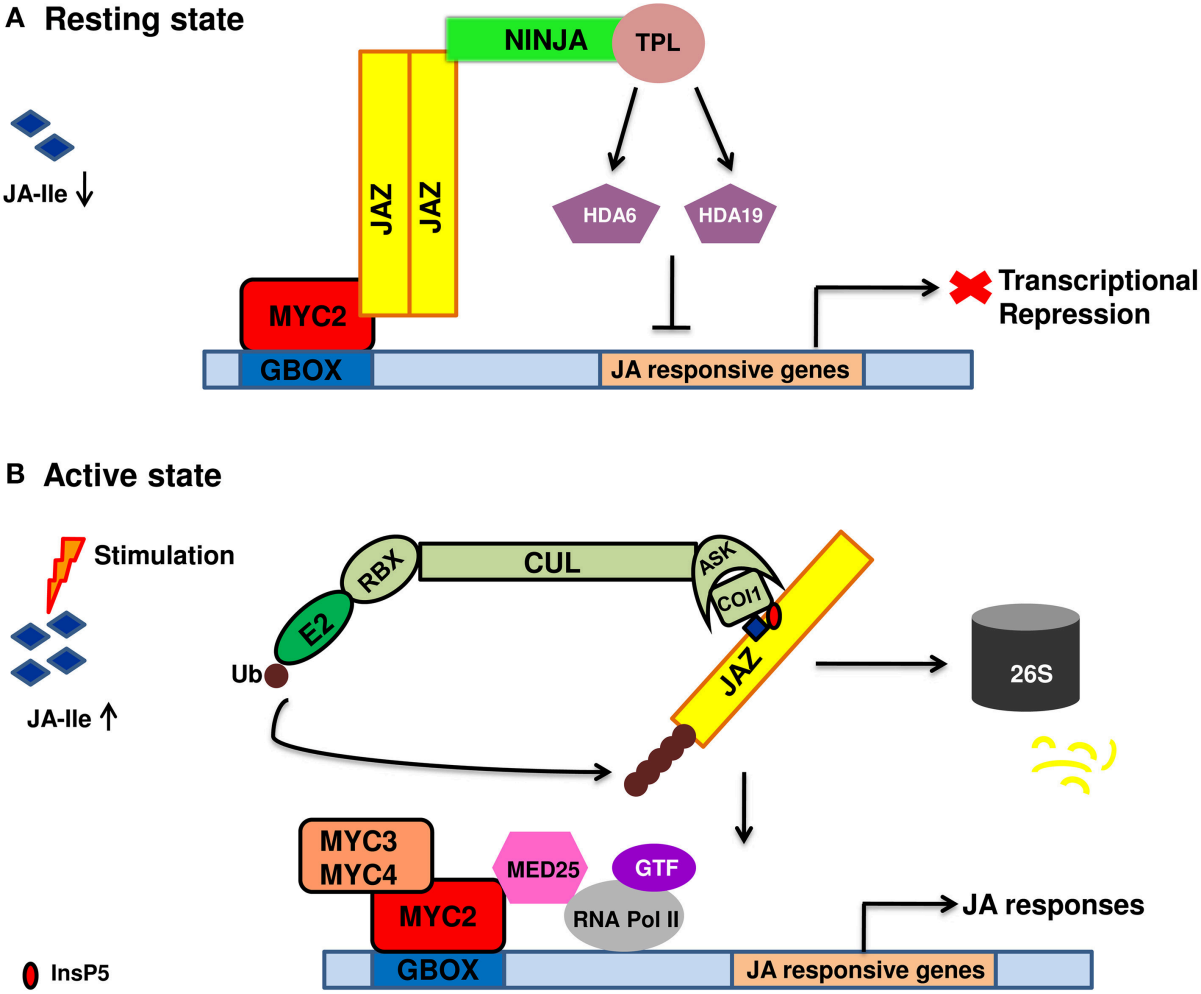
**Diagrammatic representation of jasmonic acid perception and signal transduction pathway**. **(A)** In the absence of a stimuli, jasmonic acid is not synthesized. As a result, JA mediated gene expression is inhibited due to the binding of JAZ repressors to the transcriptional activator MYC2. JAZ proteins recruit TPL and adaptor protein NINJA. Together, JAZ-NINJA-TPL form an active transcriptional repression complex that inhibit jasmonate responses by converting an open complex to a closed complex by recruiting HDA6, HDA19. **(B)** Upon stimulation by pathogen/insect/wounding, JA is rapidly synthesized and readily epimerizes to JA-Ile. It then binds to *COI1*-JAZ-InsP5 co-receptor complex causing ubiquitination and proteasomal degradation of JAZ. This frees MYC2 and its homologs from repression which then bind to G-box element present downstream of JA-responsive genes upon homo/heterodimerization. This is followed by the recruitment of MED25 that in turn bring RNAPol II and general transcription factors hence, causing diverse jasmonate responses. JA, jasmonic acid; JA-Ile, Jasmonate–isoleucine; JAZ, Jasmonate ZIM domain; NINJA, Novel adaptor of JAZ; TPL, topless; *COI1*, coronatine insensitive; Cul, Cullin1; RBX1, ring box1; Ub, ubiquitin; ASK1, *Arabidopsis* skp1 homolog 1; InsP5, inositol pentakisphosphate; GTF, general transcription factor; HDA6, HDA19, histone deacetylase 6,19; MED25, mediator25; RNAPol II, RNA polymerase II.

JA signal perception and transduction involve numerous TFs, repressors and members of ubiquitin proteasomal pathway. The section gives information of different signaling components. The current model of JA signal transduction is given in Figure 1.

### Bioactive ligand

Fonseca and co-workers in 2009 provided evidences that (+)-7-iso-JA-L-Ile is the sole natural ligand of *A. thaliana* as revealed by detailed GC-MS and HPLC analyses. Also, experiments carried out by Thines et al. (2007) revealed that only JA-Ile out of MeJA, OPDA, and JA can promote *COI1*-JAZ binding, thus confirming JA-Ile to be the direct JA signaling ligand in plants.

### SCF complex

The ubiquitin-proteasome comprises of Skp1/Cullin/F-box (SCF). Earlier, researchers believed that screening *Arabidopsis* mutants insensitive to growth inhibition with bacterial coronatine, a structural and functional homolog of JA-Ile, would result in discovering JA receptor in plants (Feys et al., 1994; Fonseca et al., 2009b). Exhaustive genetic screens identified the allele of *coronatine insensitive 1 (coi1)*, suggesting *COI1* functions in JA perception in plants. It was considered as the receptor from two lines of evidences- first, *coi1* mutant exhibits male sterility, defective responses to JA-treatment and wounding and susceptibility to necrotrophic pathogens and insects; secondly, *COI1* locus encodes an F-box protein that associates with its other counterparts SKP1, Cullin, and Rbx proteins to form an E3 ubiquitin ligase (Xie et al., 1998). *COI1* show approximately 33% sequence similarity with the auxin receptor TIR1 in amino acid sequence having leucine-rich-repeats and F-box motif (Yan et al., 2009).

### JAZ proteins

After the discovery of the receptor the most fascinating question was to find out the substrate for SCF^*COI1*^ E3 ubiquitin ligase complex. This substrate was anticipated to be the key negative regulator of JA signaling. In 2007, three independent research groups discovered a new family of protein in *Arabidopsis* called JASMONATE ZIM DOMAIN (JAZ) proteins (Chini et al., 2007; Thines et al., 2007; Yan et al., 2007). The JAZ proteins belong to the larger plant specific TIFY family, consisting of a core TIF[F/Y]XG motif within the ZN-FINGER PROTEIN EXPRESSED IN INFLORESCENCE MERISTEM (ZIM) protein domain. *A. thaliana* consists of 12 JAZ proteins (Chini et al., 2007; Thines et al., 2007; Yan et al., 2007; Chung et al., 2009) that are differentiated from other TIFY family proteins by the presence of C-terminally located Jas motif, SLX_2_FX_2_KRX_2_RX_5_PY (Nishii et al., 2000; Vanholme et al., 2007; Yan et al., 2007). They contain N-terminal domain, a highly conserved C-terminal Jas domain that mediates the interaction with the *COI1* and several transcription factors, and the conserved protein- protein interaction domain, the ZIM (TIFY) domain that helps in JAZ dimerization and interaction with NINJA (Vanholme et al., 2007; Chung et al., 2009; Pauwels and Goossens, 2011; Wasternack and Hause, 2013). The Jas domain is exclusively required to repress downstream targets of JAZ proteins (Chini et al., 2007; Thines et al., 2007; Yan et al., 2007).

The initial clue about the role of JAZ proteins in JA-signaling came from the jasmonate-*insensitive 3* mutant (*jai3*), which is a mutant of *JAI3*/*JAZ3* gene. In *jai3-1* mutant, the JAZ3 protein lacks the C-terminal portion which perturbs its binding and degradation via SCF^*COI1*^ complex. This resulted in accumulation of truncated JAI3/JAZ3 proteins in the mutant which blocked the JA-induced degradation of other JAZ proteins and hence dominant JA-insensitive phenotype (Chini et al., 2007).

### Co-receptor complex

The co-receptor complex is formed by the physical interaction of *COI1* with the Jas domain of JAZ proteins in the presence of JA-Ile (Yan et al., 2009; Sheard et al., 2010). More recently, the role of inositol pentakisphosphate (IP_5_) as a cofactor in the formation of co-receptor complex has been substantiated (Sheard et al., 2010; Mosblech et al., 2011). JA, OPDA, MeJA, and JA-Ile were tested for affinity in *COI1 JAZ1* binding. Surprisingly, only JA-Ile functioned as ligand for *COI1*-JAZ interaction (Thines et al., 2007). Based on the information available hitherto, the true jasmonates receptor is a co-receptor complex, consisting of the SCF^*COI1*^ E3 ubiquitin ligase complex, JAZ degrons (*JAZ1* to *JAZ12*) and IP_5_ (Sheard et al., 2010).

### Co-repressors

Co-repressors are transcriptional regulators that inhibit transcription initiation. One such example is the group of Groucho/Tup1 corepressor family comprising of TOPLESS (TPL) and TPL-related proteins (TPRs). TPL and TPR mediate repression by recruiting histone deacetylases and demethylases that cause chromatin modification (Macrae and Long, 2011). TPL interacts with JAZ proteins via ETHYLENE RESPONSE FACTOR (ERF)-ASSOCIATED AMPHIPHILIC REPRESSION (EAR) motif. Those JAZ proteins that do not have the repression motif recruit TPL through an adapter protein called NOVEL INTERACTOR OF JAZ (NINJA) (Pauwels et al., 2010). NINJA was first identified by Tandem affinity purification as an interactor of *JAZ1* (Pauwels et al., 2010).

### JAZ targets

The role of bHLH transcription factor MYC2 in mediating the transcriptional regulation of JA is well defined and thus has been considered the master regulator of many biological processes (Lorenzo et al., 2004; Dombrecht et al., 2007). The role of MYC2 in JA mediated responses is revealed by the study of its mutant *jasmonate-insensitive1* (*jin1*). Microarray analysis of wild type and the mutant *myc2*/*jin1* exposed the role of MYC2 in JA-dependent transcriptional regulation. MYC2 has twin function of an activator of JA-induced root growth inhibition, anthocyanin biosynthesis and oxidative stress tolerance and a repressor in mediating resistance to necrotrophic pathogens, insects and biosynthesis of tryptophan and indol glucosinolates (Lorenzo et al., 2004; Dombrecht et al., 2007). Besides MYC2, several other TFs control diverse JA response. These TFs are MYC3, MYC4, MYB, GL3, EGL3 AP, GL1 etc. (Cheng et al., 2011). MYC2 forms homo or heterodimers with its close homologs MYC3 and MYC4 to regulate the transcription of downstream targets (Fernández-Calvo et al., 2011) by binding to the G-box (5′-CAC GTG-3′) and G-box related hexamers (Abe et al., 1997; Boter et al., 2004; Yadav et al., 2005).

## Dissecting the role of jasmonates in abiotic stress

Out of 13 billion hectares of total land, only 1.6 billion is under farmland production accounting to only 12% of arable land (Syngenta, 2014)^1^. Agriculture must evolve in order to meet the demands of the increasing population. However, every year some part of the world suffers from drought, global increase in temperature, variable precipitation that eventually hampers the quality and quantity of crops. All these visible warning signs can have erratic production patterns all over the world. Plants encounter numerous challenges in terms of competition from other plants, organisms and because of the complex environment. All these provocations have made the plants tougher and more flexible. The morphological flexibility has given them the advantage to counteract, inhabit and endure biotic and abiotic challenges. Rapid changes in the plant biochemistry and physiology are mediated by the action of several phytohormones. By tradition cytokinin, auxins, brassinosteroids, and giberallins have always been associated to regulate developmental processes of plants, whereas, salicylic acid, JA and ethylene associate with plant defense and ABA regulates plant's response to abiotic stress. Now, it has been quite evident from many reports that all hormones affect multiple plant functions. Thus, one can say that hormones not only participate in plant developmental processes but also have a say in plant's response to abiotic stresses like drought, osmotic stress, chilling injury, heavy metal toxicity etc. These adversities have forced the plants to either employ avoidance as a mechanism in order to surmount the stress or choose defense over growth (Band et al., 2012; Murray et al., 2012; Petricka et al., 2012; Wasternack and Hause, 2013). Thus, stress activates signal transduction of hormones which may promote specific protective mechanisms.

### Cold stress

Among various environmental perturbations, cold stress or low temperature stress limits plant performance and geographical distribution. Cold stress can be categorized into chilling (0–15°C) and freezing that causes mayhem in tropical and subtropical plants by inducing chlorosis, necrosis, membrane damage, changes in cytoplasm viscosity, changes in enzyme activities (Ruelland and Zachowski, 2010) and ultimately death. All these physiological and biochemical changes elicit a cascade of events that cause changes in gene expression pattern and protein products and thus in due course induce plant species to adopt stratagems to tolerate low non-freezing temperatures and complete their life cycle, an experience known as cold acclimation response.

ICE-DREB1/CBF regulon plays an imperative role in cold response pathway in model plant *Arabidopsis thaliana* (Thomashow, 1999; Chinnusamy et al., 2007). *Inducer of CBF EXPRESSION 1 (ICE1)* is a MYC-type transcription factor that acts as a master regulator and controls *CBF/DREB1* pathway. In Arabidopsis, three *CBF/DREB1* are involved in the regulation of *COLD REGULATED (COR)* gene expression and tolerance to cold stress (Gilmour et al., 2000, 2004). In this pathway, *ICE1* positively regulates and activates *CBF/DREB1* genes that encode AP2/ ERF type TF family. CBFs, by binding to C-repeat *(CRT)* element induce COR genes leading to tolerance to cold stress (Thomashow, 2010).

Tropical and subtropical fruits like mango, avocado, papaya etc. exhibit symptoms due to chilling injuries such as browning discoloration and off-flavor in the fruit. Previous studies have specified the role of MeJA in alleviating chilling injury by inducing the production of cryo-protective agents, proteinase inhibitors, polyamines, ABA, lower activity of LOXs, and antioxidants (Wang and Buta, 1994; González-Aguilar et al., 2000; Cao et al., 2009; Zhao et al., 2013). Role of MeJA in response to freezing tolerance has also been studied in rice seedlings. It has been reported that rather than treating rice with MeJA during or after stress imposition, MeJA treatment before chilling remarkably enhances the survival ratio of chilled rice seedlings (Lee et al., 1997). Furthermore, it was observed that MeJA maintained the well-watered status of chilled plants by preventing stomatal opening and enhancing hydrolytic conductivity.

Du et al. (2013) reported an increase in the level of endogenous JA on exposure to cold stress. Microarray analysis of cold treated rice seedlings revealed upregulation of JA biosynthesis genes *OsDAD1, OsLOX2, OsAOC, OsAOS1, OsAOS2, OsOPR1*, and *OsOPR7*. Besides this, JA signaling genes such as *OsJAR1, OsbHLH148*, and *OsCOI1a* showed up regulation upon cold exposure. Furthermore, positive role of jasmonate in enhancing constitutive and cold acclimation–induced freezing tolerance of *Arabidopsis* has been reported by Hu et al. (2013). Treatment of WT *Arabidopsis* seedlings with exogenous methyl jasmonate improved the endurance and plant freezing tolerance. On the contrary, blocking JA biosynthesis and signaling pathway rendered the plant hypersensitive to freezing stress.

Of lately, role of JAZ repressors in controlling cold stress tolerance has also been investigated. Under normal growth conditions *Arabidopsis JAZ1* and *JAZ4* interact and suppress cold TFs *ICE1* and *ICE2*, thus quelling *ICE1-CBF/DREB1* pathway (Hu et al., 2013). Also, over expression of *JAZ1* or *JAZ4* repressed the cold-induced expression of *CBF/DREB1*, in that way contributing the transgenic plants sensitive to freezing. However, over expression of *ICE1* was able to salvage freezing sensitive phenotype of *coi1-1* mutant plants.

Recent studies have suggested the role of downstream transcription factors in the regulation of cold responses. Homologs of Arabidopsis MYC2 TF have been isolated and characterized in *Musa accuminata* (Peng et al., 2013; Zhao et al., 2013). *MaMYC2a* and *MaMYC2b* have been shown to be rapidly induced by MeJA treatment upon cold exposure. Expression profiles of CBF cold responsive pathway genes, including *MaCBF1, MaCBF2, MaCOR1, MaKIN2, MaRD2*, and *MaRD5* demonstrated the induction of CBF genes by MeJA upon cold stress. Also, they have been accounted to physically interact with *MaICE1*, therefore suggesting a potential cross talk between two signal transduction pathways.

*Arabidopsis* SENSITIVE TO FREEZING 6 (SFR6) controls cold regulated gene expression and is well known to act post-translationally on the CBF module (Knight et al., 1999, 2009; Boyce et al., 2003). Additionally, it is involved in regulating JA responses (Wathugala et al., 2012; Zhang et al., 2012). Very recently, SFR6 has been identified as the MEDIATOR16 of the plant mediator complex that is involved in recruiting RNA polymerase II to promoters carrying CRT/DREB motif (Hemsley et al., 2014).

Similar to JA, salicylic acid (SA) is a powerful tool in regulating cold stress tolerance. Exogenous application of SA on *H. vulgare* genotypes resulted in cold tolerance by enhancing antioxidant enzymes, ice nucleation activity and the patterns of apoplastic proteins (Mutlu et al., 2013). Accumulation of endogenous free SA and glucosyl SA has been reported during chilling in *Arabidopsis* shoots, wheat and grape berry (Scott et al., 2004; Wan et al., 2009; Kosová et al., 2012). However, it has been observed that concentration and duration of applied SA greatly influence its utility. High concentration and continual application of SA decreases the cold tolerance capacity of plants as observed in some *Arabidopsis* mutants, such as *CONSTITUTIVE EXPRESSER OF PATHOGENESIS-RELATED GENE1 (CPR1) AND ACCELERATED CELL DEATH6 (ACD6)*, in which SA is over-accumulated, exhibit a dwarf phenotype and freezing sensitivity (Scott et al., 2004; Miura et al., 2010).

Fung et al. (2004) reported an alleviation of chilling injury in freshly harvested green bell pepper (*Capsicum annuum*) by methyl SA (MeSA) and MeJA vapors. This reduction of chilling injury in the green bell pepper was related with an increase in the expression of the *ALTERNATIVE OXIDASE* (*AOX*) gene induced by MeSA and MeJA vapors. Feng et al. (2008) later reported that the expression of *AOX* was enhanced under chilling stress. It has been earlier reported that AOX expression increased in response to low temperature stresses in rice (Ito et al., 1997). All these observations suggest that AOX is involved in combating cold stress. Siboza et al. (2014) reported that combined treatment of MeJA and SA reduced ROS accumulation, lipid peroxidation and increased chilling tolerance in lemon during cold storage by increasing the synthesis of total phenolics and phenylalanine ammonia lyase (PAL) and inhibiting the activity of polyphenol oxidase (PPO) and peroxidase (POD). Induction of PAL activity has been considered a good marker of CI (Martínez-Téllez and Lafuente, 1993; Sanchez-Ballesta et al., 2000).

Findings by Miura and Ohta (2010) have indicated *ICE1* to be an essential integrator of SA signaling and cold response pathway. *ice1* mutant also showed an up-regulation of SA-inducible genes. Additionally, *CALMODULIN BINDING TRANSCRIPTION ACTIVATOR 3 CAMTA3/AtSR1* has been shown to participate in enhancing cold tolerance by binding to the promoter of *CBF1* and *CBF2/DREB1C* (Doherty et al., 2009). Recently, Kim et al. (2013) extended the findings by showing the up-regulation of 15% cold inducible genes by *CAMTA* TFs. It has been established that *CAMTA3* behaves as a repressor of SA biosynthesis at warm temperature under non-stressed condition (Du et al., 2009). But, under cold conditions this repression is overcome as reported by Kim and co-workers in 2013, leading to an increase in the level of SA and up regulation of SA responsive genes. However, the results indicate that SA does not contribute to freezing tolerance but genes induced by SA at low temperature increase the resistance to pathogen attack. The above findings raise the possibility that *CAMTA3* does not only cause changes in gene expression at low temperature but also plays a role in regulating genes involved in SA biosynthesis at low temperature.

**Figure 2:**
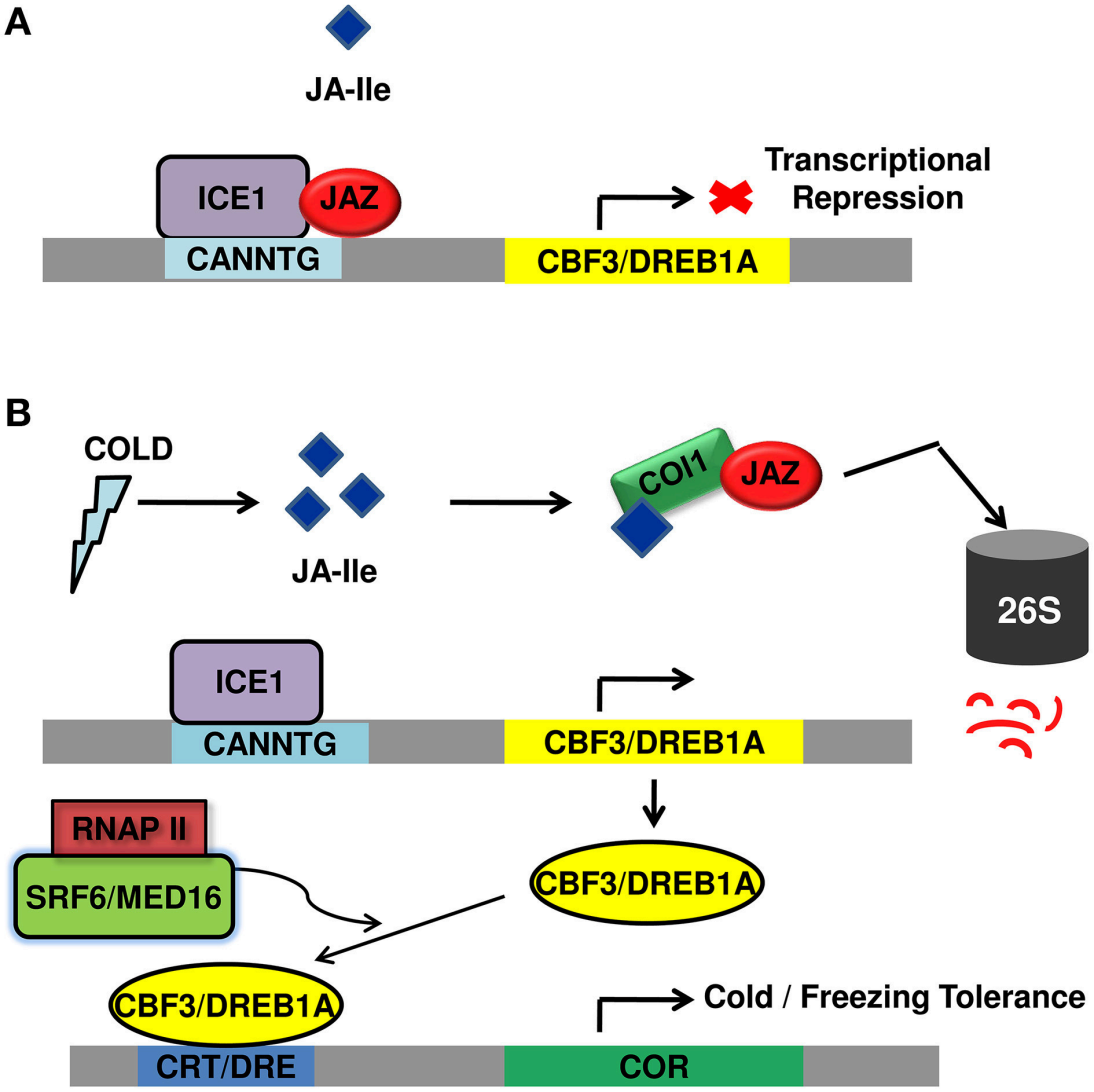
**Diagrammatic representation of regulation of cold stress tolerance by JA signal transduction pathway**. **(A)** Under normal growth conditions, JAZ repressor proteins physically interact and suppress cold TF *ICE1*, thus repressing ICE/CBF-DREB1 pathway and rendering plants sensitive to freezing. **(B)** Upon cold induction, JA is synthesized that rapidly isomerizes to JA-Ile and lead to proteasomal degradation of JAZ. This frees *ICE1* that binds to CBF3 responsive element leading to its expression. The CBF proteins bind to CRT/DRE element causing the expression of COR genes that participate in cold/freezing tolerance. JAZ, jasmonate ZIM domain protein; TF, transcription factor; *ICE1*, inducer of CBF expression; *CBF-DREB1*, C repeat binding factor 1-dehydartion responsive element binding factor1B; COR, cold regulated; DRE, dehydration responsive element; CRT, C-repeat; SFR6/MED16, sensitive to freezing 6/Mediator 16; RNAPII, RNA polymerase II; *COI1*, coronatine insensitive.

Unlike the close interaction between components of JA signaling and cold regulated transcription factors and JA functioning as a crucial upstream signal to *ICE-CBF/DREB1* pathway (Figure 2), very few reports are available on the molecular mechanisms underlying SA-mediated improved plant tolerance to cold temperature. However, the above reports shed light upon the relatedness between cold signaling and SA signaling. Moreover, the participation of *ICE1* in both JA and SA signaling pathway, ROS avoidance mechanism employed both by SA and JA points out the possible crosstalk between JA and SA signal transduction pathway to fight cold stress.

Earlier work conducted by Wilen et al. (1993) pointed out synergism between ABA and JA in inducing freezing tolerance in bromegrass cell cultures. The role of JAs in imparting freezing stress tolerance via *CBF1/DREB1* pathway is well known. To further investigate their role in imparting freezing tolerance via *CBF/DREB1* independent pathway, Hu et al. (2013) carried out microarray and real time analysis in WT and *coi1* mutant plants upon cold treatment. They observed that many cold responsive genes that do not fall in *CBF1/DREB1* pathway were down-regulated in *coi1* mutant plants, thus indicating that jasmonate might play a role in imparting freezing tolerance via *CBF/DREB1* independent pathway. To further assess whether JAs affect all cold regulated expression of genes, Hu and co-workers carried out expression analysis of cold responsive genes falling in ABA pathway in WT and *coi1* plants and it was observed that the transcript level in *coi1* mutant plants changed similar to WT upon cold treatment. Hence, cold tolerance may be provided independently via ABA and JA signaling pathways.

Like jasmonates, ethylene (ET) has been demonstrated to modulate various abiotic stress responses. Based on findings from Arabidopsis, ET signaling pathway has been shown to negatively regulate freezing stress responses (Shi et al., 2012). EIN3, a positive regulator of ET signaling pathway transcriptionally represses the CBF pathway and thus acts antagonist to JA. Furthermore, Zhu and co-workers in 2011 revealed a crosstalk between JA and ET signaling pathways through the interaction between JAZ and their targets EIN3/EIL1 (Zhu et al., 2011). According to their model, ET is required for stabilization of EIN3/EIL1 and JA is needed for their release from JAZ degradation, as speculated by them. This dual regulation balances the detrimental effects on plant growth and development and establishes appropriate stress tolerance, as speculated by them. Thus, one can say that the CBF signaling pathway is regulated at multiple levels by JAs as it positively regulates freezing tolerance and at the same time represses the *CBF/DREB1* pathway via EIN3/EIL1.

The role of JAs and its interplay with other phytohormones to modulate cold stress responses has been demonstrated in this section. The interaction between different components for example JAZ with EIN3/EIL1 or JAZ with *ICE1*/*ICE2* mediates ET, JA, cold signal transduction pathways. Identifying the novel components involved in crosstalk between JA and other hormones in regulating CBF cold response pathway would help in dissecting the exact molecular mechanism.

### Heat stress

Temperature above the optimum for growth can be detrimental, causing injury or irreversible damage to plant growth and development. IPCC (2014) reports suggest an approximate increase of 4–5°C in the average temperature by the end of the twenty-first century. High temperature stress negatively influences plant processes and cellular machinery, thereby impairing cell homeostasis (Bokszczanin et al., 2013). Plants have the inherent ability to ameliorate the adverse effects of heat shock by the phenomenon of basal thermotolerance whereas, acquired thermotolerance is achieved when plants are pre-exposed to high, non-lethal temperature (Bokszczanin et al., 2013). In response to high temperature, plants synthesize HEAT SHOCK PROTEINS (HSPs) that prevent denaturation and assist refolding of damaged proteins (Boston et al., 1996).

The role of JA signaling in contributing to thermotolerance has been recently established in WT *Arabidopsis* (Clarke et al., 2009). Exogenous application of low concentration of MeJA maintained cell viability in heat stressed plants as demonstrated by electrolyte leakage assays. Moreover, heating WT Arabidopsis led to the accumulation of several jasmonates including OPDA, MeJA, JA, and JA-Ile. But, no evidence was found that thermotolerance conferred by MeJA elicited HSP gene expression. Expression level of jasmonate inducible gene PDF1.2 was found to be high upon heat stress exposure. The final proof of the role of jasmonates in imparting thermotolerance was confirmed by mutant analysis wherein JA and SA signaling mutants *coi1-1, opr3, and jar1-1cpr5-1* were found to be sensitive to heat stress (Clarke et al., 2009). Thus, establishing the fact that both SA and JA provide basal thermotolerance.

SUPPRESSOR OF G2 ALLELE OF SKP1 (SGT1) protein operates as a cofactor of HEAT SHOCK PROTEIN 90 (HSP90) in both plants and mammals forming functional complexes and providing thermotolerance. Intensive genetic and biochemical screening confirmed the role of SGT1 in plant hormone signaling pathways that involve F-box proteins and ubiquitin ligases such as *COI1* of JA signaling. SGT1 maintains the steady state of *COI1* (Zhang et al., 2015). Reduced transcript levels of JA marker genes in HSP90 RNAi lines confirmed the crucial role played by HSP90 and HSP70 in JA-COR (coronatine) responses. Additionally, pre-treating WT *Arabidopsis* with HSP inhibitor attenuated COR (coronatine) triggered gene expression. Given the facts that HSP proteins have numerous substrates including transcription factors, E3 ligases, kinases and the above data suggested that *COI1* is a client protein of SGT1b–HSP70–HSP90 chaperone complexes. This widens the functional capacity of SGT1b–HSP70–HSP90 chaperone complexes in regulating JA responses.

WRKY super family consists of 74 members in *Arabidopsis thaliana* (Eulgem and Somssich, 2007) and is subdivided into three groups based on the number of WRKY domains and the features of their Zn finger like motifs (Eulgem et al., 2000). They are a group of regulatory proteins that participate in plant developmental processes but notably in a plethora of biotic and abiotic challenges. There are sufficient evidences that many WRKY genes participate in abiotic stresses, including heat stress. Several WRKY TFs have been revealed to impart thermotolerance like *AtWRKY25* (Zhu et al., 2009), *AtWRKY39* (Li et al., 2010), and *OsWRKY11* (Wu et al., 2009). Dang et al. (2013) showed that *CaWRKY40* was involved in heat stress and was transcriptionally induced by exogenous application of JA. Moreover, over expression lines of *CaWRKY40* derepressed JA biosynthesis *NtLOX1* by heat stress. Together, the findings suggest that JA mediates the expression of *CaWRKY40* leading to the expression of downstream thermotolerance-related genes.

JA and ET act as antagonists in regulating heat stress responses. Studies by Clarke et al. (2009) showed that despite being produced in response to heat stress, ET negatively regulates heat stress tolerance. They found out that *ein2* mutant displayed thermotolerance, hence suggesting that EIN2 mediated pathway negatively regulates thermotolerance. They also demonstrated that ET production was augmented by JA from studies carried out in WT and *opr3* mutant.

## Concluding remarks

The daunting issue of nutritional and food security has resulted in a quest among researchers to elucidate the action of phytohormones in stress related responses. Tolerance to (a)biotic stresses is a challenge of agro-economic impact. For this, a model eudicot, *Arabidopsis* has emerged as a quintessential system to study plant stress tolerance. However, crop plants are exposed to complex environmental perturbations in the field. Therefore, thorough research will be required to understand how crops respond to multiple abiotic stresses in order to develop new varieties that can withstand global climate changes.

The role of jasmonates in plant development is very well established (Wasternack and Hause, 2013). Nevertheless, a significant body of research suggests the role of jasmonic acid in plant responses to abiotic stresses. Growing sagacity of crosstalk between JAs and other hormones and with different components of abiotic signal transduction may allow us to dissect key factors involved in the crosstalk. Recent reports suggest interplay between cold regulated transcription factors and components of JA biosynthesis and signaling. The interaction of JAZ repressors with transcriptional activators *ICE1* and *ICE2* and falling upstream to the cold response pathway (Hu et al., 2013) as well as the induction of JA genes upon cold stimulus (Du et al., 2013; Hu et al., 2013) demonstrates the sharing of common signaling components and hence a closed interaction between the two signal transduction pathway. Additionally, several lines of evidences suggest the role of JAs in imparting thermotolerance. In addition, mutant analyses have also confirmed the role of JAs in heat tolerance responses. Modifying different upstream/downstream signaling components by genetic engineering can improve the adaptability of plants in response to temperature stress. However, there is still obscurity in the crosstalk among different signaling pathways. The application of forward and reverse genetic analysis in model plants along with the genomics and proteomics tools will help us in the discovery of new regulatory components and in dissecting the complex interactions between different signaling pathways to elucidate hormone action in stress related context.

### Conflict of interest statement

The authors declare that the research was conducted in the absence of any commercial or financial relationships that could be construed as a potential conflict of interest.

## Footnotes

^1^Syngenta (2014). Our industry 2014.
